# Supplementary material for: Modeling glioblastoma heterogeneity as a dynamic network of cell states
Source: Mol Syst Biol. 2021 Sep 16;17(9):e10105. doi: 10.15252/msb.202010105 (PMC8444284; doi:10.15252/msb.202010105)
Supplement: Supplementary file 5 — Source Data for Figure 3 [file MSB-17-e10105-s001.zip › Figure3A_sourcedata/GSEA_3065/nosigdb_state3.GseaPreranked.1622718426028/LARSSON_STATE_1.html]

Details for gene set LARSSON\_STATE\_1[GSEA]

|  || Dataset | state3 |
| Phenotype | NoPhenotypeAvailable |
| Upregulated in class | na\_neg |
| GeneSet | LARSSON\_STATE\_1 |
| Enrichment Score (ES) | -0.8267737 |
| Normalized Enrichment Score (NES) | -2.9152124 |
| Nominal p-value | 0.0 |
| FDR q-value | 0.0 |
| FWER p-Value | 0.0 |
Table: GSEA Results Summary

  

Fig 1: Enrichment plot: LARSSON\_STATE\_1      
 Profile of the Running ES Score & Positions of GeneSet Members on the Rank Ordered List

  

| PROBE | GENE SYMBOL | GENE\_TITLE | RANK IN GENE LIST | RANK METRIC SCORE | RUNNING ES | CORE ENRICHMENT || 1 | POSTN |  |  | 24 | 0.487 | 0.0134 | No |
| 2 | CTSC |  |  | 226 | 0.272 | -0.0008 | No |
| 3 | DCBLD2 |  |  | 243 | 0.265 | 0.0061 | No |
| 4 | SCG2 |  |  | 778 | 0.160 | -0.0503 | No |
| 5 | PTHLH |  |  | 854 | 0.152 | -0.0539 | No |
| 6 | SLC14A1 |  |  | 1130 | 0.127 | -0.0814 | No |
| 7 | SPP1 |  |  | 1358 | 0.111 | -0.1040 | No |
| 8 | ANK3 |  |  | 1702 | 0.091 | -0.1406 | No |
| 9 | IGFBP3 |  |  | 2112 | 0.071 | -0.1855 | No |
| 10 | IGFBP5 |  |  | 3555 | 0.027 | -0.3512 | No |
| 11 | CTSV |  |  | 4007 | 0.015 | -0.4028 | No |
| 12 | CCK |  |  | 4463 | 0.002 | -0.4553 | No |
| 13 | PLP2 |  |  | 4543 | 0.000 | -0.4644 | No |
| 14 | TMEFF2 |  |  | 4708 | -0.004 | -0.4833 | No |
| 15 | SYNJ2 |  |  | 5836 | -0.034 | -0.6123 | No |
| 16 | USP53 |  |  | 5858 | -0.035 | -0.6136 | No |
| 17 | TXNRD1 |  |  | 6009 | -0.040 | -0.6296 | No |
| 18 | DKK1 |  |  | 6057 | -0.041 | -0.6337 | No |
| 19 | TUBB4B |  |  | 6059 | -0.042 | -0.6324 | No |
| 20 | CAV1 |  |  | 6859 | -0.073 | -0.7223 | No |
| 21 | VIPR1 |  |  | 6987 | -0.080 | -0.7343 | No |
| 22 | ANXA1 |  |  | 7032 | -0.083 | -0.7367 | No |
| 23 | PYGB |  |  | 7034 | -0.083 | -0.7340 | No |
| 24 | CLIC1 |  |  | 7041 | -0.084 | -0.7319 | No |
| 25 | LMNA |  |  | 7412 | -0.109 | -0.7711 | No |
| 26 | CCND1 |  |  | 7456 | -0.113 | -0.7723 | No |
| 27 | TWF2 |  |  | 7562 | -0.123 | -0.7803 | No |
| 28 | CAST |  |  | 7828 | -0.155 | -0.8058 | No |
| 29 | CAPN2 |  |  | 7878 | -0.162 | -0.8061 | No |
| 30 | WDR1 |  |  | 8058 | -0.195 | -0.8203 | Yes |
| 31 | EIF6 |  |  | 8066 | -0.197 | -0.8146 | Yes |
| 32 | PLIN3 |  |  | 8092 | -0.202 | -0.8108 | Yes |
| 33 | FBXO32 |  |  | 8117 | -0.208 | -0.8067 | Yes |
| 34 | NETO2 |  |  | 8118 | -0.208 | -0.7998 | Yes |
| 35 | PMAIP1 |  |  | 8157 | -0.217 | -0.7970 | Yes |
| 36 | ANXA2 |  |  | 8206 | -0.230 | -0.7949 | Yes |
| 37 | CAVIN1 |  |  | 8208 | -0.231 | -0.7874 | Yes |
| 38 | TWIST1 |  |  | 8248 | -0.239 | -0.7840 | Yes |
| 39 | HOXB2 |  |  | 8249 | -0.239 | -0.7760 | Yes |
| 40 | NQO1 |  |  | 8263 | -0.244 | -0.7695 | Yes |
| 41 | PDLIM4 |  |  | 8272 | -0.246 | -0.7622 | Yes |
| 42 | UPP1 |  |  | 8293 | -0.256 | -0.7560 | Yes |
| 43 | MAP3K7CL |  |  | 8298 | -0.258 | -0.7480 | Yes |
| 44 | STK17A |  |  | 8300 | -0.258 | -0.7395 | Yes |
| 45 | CCDC85B |  |  | 8332 | -0.266 | -0.7343 | Yes |
| 46 | GADD45GIP1 |  |  | 8338 | -0.267 | -0.7260 | Yes |
| 47 | PTTG1 |  |  | 8366 | -0.279 | -0.7199 | Yes |
| 48 | ARPC5 |  |  | 8367 | -0.279 | -0.7106 | Yes |
| 49 | GCHFR |  |  | 8371 | -0.280 | -0.7017 | Yes |
| 50 | ACTG1 |  |  | 8389 | -0.289 | -0.6941 | Yes |
| 51 | DPYSL3 |  |  | 8391 | -0.291 | -0.6845 | Yes |
| 52 | CTNNBIP1 |  |  | 8409 | -0.299 | -0.6766 | Yes |
| 53 | PRDX6 |  |  | 8410 | -0.300 | -0.6667 | Yes |
| 54 | PKM |  |  | 8420 | -0.301 | -0.6577 | Yes |
| 55 | ACTB |  |  | 8444 | -0.312 | -0.6500 | Yes |
| 56 | COTL1 |  |  | 8449 | -0.314 | -0.6401 | Yes |
| 57 | CDC42EP3 |  |  | 8473 | -0.326 | -0.6319 | Yes |
| 58 | TUBA1C |  |  | 8478 | -0.328 | -0.6215 | Yes |
| 59 | S100A11 |  |  | 8490 | -0.336 | -0.6116 | Yes |
| 60 | MYL6 |  |  | 8495 | -0.339 | -0.6009 | Yes |
| 61 | DRAP1 |  |  | 8498 | -0.340 | -0.5898 | Yes |
| 62 | HMGA1 |  |  | 8519 | -0.346 | -0.5807 | Yes |
| 63 | PMEPA1 |  |  | 8520 | -0.346 | -0.5692 | Yes |
| 64 | MMP24OS |  |  | 8537 | -0.356 | -0.5592 | Yes |
| 65 | C12orf75 |  |  | 8548 | -0.363 | -0.5484 | Yes |
| 66 | DSTN |  |  | 8559 | -0.371 | -0.5372 | Yes |
| 67 | AP1S2 |  |  | 8570 | -0.382 | -0.5257 | Yes |
| 68 | ODC1 |  |  | 8576 | -0.385 | -0.5135 | Yes |
| 69 | TMEM158 |  |  | 8586 | -0.391 | -0.5016 | Yes |
| 70 | CSRP2 |  |  | 8589 | -0.393 | -0.4888 | Yes |
| 71 | TPM4 |  |  | 8594 | -0.396 | -0.4761 | Yes |
| 72 | RPL8 |  |  | 8602 | -0.405 | -0.4635 | Yes |
| 73 | SMS |  |  | 8624 | -0.430 | -0.4517 | Yes |
| 74 | GAP43 |  |  | 8637 | -0.441 | -0.4385 | Yes |
| 75 | S100A13 |  |  | 8652 | -0.457 | -0.4249 | Yes |
| 76 | UBE2S |  |  | 8655 | -0.458 | -0.4100 | Yes |
| 77 | ARL4C |  |  | 8660 | -0.467 | -0.3950 | Yes |
| 78 | TXN |  |  | 8662 | -0.467 | -0.3796 | Yes |
| 79 | PHLDA1 |  |  | 8670 | -0.476 | -0.3646 | Yes |
| 80 | PPP1R14B |  |  | 8674 | -0.484 | -0.3489 | Yes |
| 81 | OAZ1 |  |  | 8678 | -0.491 | -0.3330 | Yes |
| 82 | ARPC1A |  |  | 8680 | -0.493 | -0.3168 | Yes |
| 83 | PRELID1 |  |  | 8684 | -0.498 | -0.3006 | Yes |
| 84 | SPATS2L |  |  | 8691 | -0.509 | -0.2844 | Yes |
| 85 | SRM |  |  | 8693 | -0.511 | -0.2676 | Yes |
| 86 | TPI1 |  |  | 8709 | -0.538 | -0.2515 | Yes |
| 87 | HINT1 |  |  | 8712 | -0.547 | -0.2335 | Yes |
| 88 | S100A16 |  |  | 8714 | -0.555 | -0.2153 | Yes |
| 89 | S100A10 |  |  | 8717 | -0.558 | -0.1970 | Yes |
| 90 | S100A6 |  |  | 8729 | -0.615 | -0.1779 | Yes |
| 91 | PFN1 |  |  | 8732 | -0.624 | -0.1574 | Yes |
| 92 | RPSA |  |  | 8736 | -0.657 | -0.1360 | Yes |
| 93 | LGALS1 |  |  | 8744 | -0.718 | -0.1130 | Yes |
| 94 | SH3BGRL3 |  |  | 8746 | -0.785 | -0.0871 | Yes |
| 95 | RPS12 |  |  | 8747 | -0.792 | -0.0608 | Yes |
| 96 | TMSB4X |  |  | 8748 | -0.920 | -0.0303 | Yes |
| 97 | TMSB10 |  |  | 8749 | -0.922 | 0.0002 | Yes |
Table: GSEA details [plain text format]

  

Fig 2: LARSSON\_STATE\_1: Random ES distribution      
 Gene set null distribution of ES for **LARSSON\_STATE\_1**
